# Long noncoding RNA FOXD2‐AS1 aggravates hepatocellular carcinoma tumorigenesis by regulating the miR‐206/MAP3K1 axis

**DOI:** 10.1002/cam4.3204

**Published:** 2020-06-18

**Authors:** Wei Hu, Hui Feng, Xiaoyu Xu, Xin Huang, Xingyue Huang, Wenwei Chen, Lidan Hao, Wenfang Xia

**Affiliations:** ^1^ Department of Gynecology and Obstetrics Ultrasound Renmin Hospital of Wuhan University Wuhan China; ^2^ Department of Administration Office Renmin Hospital of Wuhan University Wuhan China; ^3^ Department of Obstetrics East Hospital of Renmin Hospital of Wuhan University Wuhan China; ^4^ Department of Ultrasound Renmin Hospital of Wuhan University Wuhan China; ^5^ Department of Critical Care Medicine Renmin Hospital of Wuhan University Wuhan China

**Keywords:** FOXD2‐AS1, hepatocellular carcinoma, MAP3K1, miR‐206

## Abstract

LncRNAs play crucial roles in the development of various cancers including hepatocellular carcinoma (HCC). Nevertheless, the function of the long noncoding RNA (lncRNA) FOXD2‐AS1 in HCC is still poorly understood. In this study, we focused on the role of FOXD2‐AS1 in HCC. We found that FOXD2‐AS1 was significantly upregulated in HCC cells in comparison to normal human liver cells, LO2. In this study, we also demonstrated that miR‐206 expression was greatly reduced in HCC cells. Furthermore, the inhibition of FOXD2‐AS1 repressed HCC cell proliferation, enhanced cell apoptosis, and restrained cell invasion and migration. The knockdown of FOXD2‐AS1 elevated miR‐206 expression, and we validated an interaction between these RNAs. Additionally, miR‐206 mimics inhibited HCC development while miR‐206 mimics had the opposite effect. MAP kinase 1 (MAP3K1) was predicted to be a target of miR‐206. We discovered that FOXD2‐AS1 modulated MAP3K1 expression by sponging miR‐206 in MHCC‐97L and HepG2 cells. Finally, our in vivo experiments validated that the knockdown of FOXD2‐AS1 inhibited HCC progression by modulating the miR‐206/MAP3K1 axis. In conclusion, this work implies FOXD2‐AS1 accelerates HCC progression through sponging miR‐206 and regulating MAP3K1 expression.

## Graphical abstract

FOXD2‐AS1 was significantly upregulated in HCC cells. FOXD2‐AS1 can regulate MAP3K1 by acting as a sponge of miR‐206. FOXD2‐AS1 can serve as a tumor oncogenic role in HCC via interacting with miR‐206 and MAP3K1.

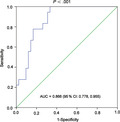

## 1 INTRODUCTION

Hepatocellular carcinoma (HCC) is a leading cause of cancer‐related death across the world.^1^, ^2^ The incidence of HCC is over half a million annually.^3^ Despite the significant advances that have been made in available treatments, the prognosis of HCC is still unsatisfied.^4^, ^5^ To develop effective treatments, it is important to determine the cellular mechanisms that underly HCC development.

LncRNAs are a class of RNA transcripts with more than 200 nucleotides and no protein encoding potential.^6^ Accumulating evidence indicates lncRNAs are closely associated with multiple biological processes^7^ and are implicated in numerous cancers. For example, lncRNA TUG1 is elevated in HCC, which regulates HCC cell growth and apoptosis by repressing KLF2.^8^ LncRNA UFC1 can interact with HuR and increase β‐catenin levels in HCC cells.^9^ Additionally, lncRNA HULC can modulate lipid metabolism in hepatoma cells through targeting RXRA.^10^ LncRNA DANCR promotes the stemness of HCC cells by repressing CTNNB1.^11^ Previous studies have reported that FOXD2‐AS1 lncRNA can serve as a significant oncogene in several cancers, including lung cancer and gastric cancer.^12^, ^13^ Moreover, a recent study demonstrated FOXD2‐AS1 as a prognostic biomarker for HCC.^14^ However, the functional role and the underlying mechanism of FOXD2‐AS1 in HCC development remain unknown.

MicroRNAs are small RNAs involved in many pathological processes through regulating target genes.^15^, ^16^, ^17^ Studies have well established microRNA as crucial participators in HCC. For instance, miR‐195 can inhibit HCC metastasis by inhibiting YAP.^18^ Decreased miR‐125a level can indicate poor survival of HCC patients.^19^ miR‐30a‐5p can suppress HCC proliferation and invasion by targeting FOXA1.^20^ miR‐18a can trigger HCC proliferation and metastasis by targeting KLF4.^21^ Previous studies have found that miR‐206 can act as a tumor suppressor in multiple cancers. It can repress renal cell cancer growth by targeting GAK,^22^ and suppress glioblastoma progression by regulating BCL‐2.^23^ In HCC, the expression of miR‐206 is significantly reduced, and it is also a potential diagnostic biomarker.^24^ However, the potential function of miR‐206 in HCC remains poorly understood.

We found that FOXD2‐AS1 expression was upregulated in HCC cells. Depletion of FOXD2‐AS1 restrained HCC cell growth. Meanwhile, miR‐206 was predicted as a target of FOXD2‐AS1, and MAP3K1 was predicted as a target of miR‐206. Thus, we investigated the functional relationship of FOXD2‐AS1, miR‐206, and MAP3K1, and hypothesized that FOXD2‐AS1 can regulate HCC progression by modulating miR‐206 and MAP3K1 expression.

## 2 MATERIALS AND METHODS

### 2.1 Clinical samples

Clinical samples were obtained from HCC patients at Renmin Hospital of Wuhan University. All subjects provided written informed consent. The study was approved by the Institutional Ethics Committee of Renmin Hospital of Wuhan University and conducted according to the Helsinki Declaration and government policies. Blood samples were disposed within 2 hours. Then, serum was obtained by centrifugation (1200 × *g*, 10 minutes) at 4°C. We maintained the serum samples in liquid nitrogen for RNA extraction.

### 2.2 Cell culture

HepG2, MHCC‐97L, MHCC‐97H, SNU449, LO2, and HEK‐293T cells were purchased from ATCC (Manassas, VA, USA) and cultivated in DMEM medium (GIBCO, Carlsbad, CA, USA) in a 5% CO_2_ incubator at 37°C. The medium was added with 10% heat‐inactivated FBS (GIBCO, Carlsbad, CA, USA), 100 U/mL penicillin, and 100 mg/mL streptomycin.

### 2.3 Lentiviral transfection

Full‐length FOXD2‐AS1 cDNA was amplified from HCC cells. Sequences were constructed with shLuc as a negative control (NC). Short hairpin RNAs (shRNA) competing against FOXD2‐AS1 were designed by Shanghai GenePharma Co., Ltd. shRNAs were subcloned into lentiviral plasmids and infected with lentiviral packaging plasmids to produce two lentiviruses. Cells with FOXD2‐AS1 downregulation were incubated with 800 μg/mL G418 at a multiplicity of infection (MOI) of 1‐3. Cells were selected 48 hours later by cultivation in a culture medium supplemented with 10 μg/mL blasticidin for 7 days. Surviving cells were reseeded using fresh culture medium.

### 2.4 CCK8 assay

Cells were grown onto a 96‐well plate. Next, cells were infected with LV‐shFOXD2‐AS1 or LV‐NC for 48 hours. Then, 100 μL CCK8 reagent (Dojindo Molecular Technologies, Tokyo, Japan) was added. A microplate reader (Bio‐Tek) was used to record CCK8 measurements.

### 2.5 EdU assay

EdU proliferation experiments were performed with a EdU kit (Millipore, Billerica, MA, US) to assess cell proliferation. Cells were incubated with 50 μmol/L EdU for 2 hours, and EdU‐positive cells were observed using Apollo and DAPI staining.

### 2.6 Apoptosis

The PI/Annexin V‐FITC kit (BioLegend) was used for double apoptotic staining. All cells were digested using trypsin. The cells were then centrifuged, collected, and washed with cold PBS. PI and Annexin V‐FITC (5 µL each) were used and the cells were incubated for 15 minutes without light. Samples were analyzed using a BD FACS Calibur Cytometer (BD Bioscience).

### 2.7 Transwell migration and invasion assay

For the Transwell migration assay, 1 × 10^5^/mL cells were added to the Transwell upper chambers without Matrigel. To evaluate cell invasion, Matrigel‐coated Transwell chambers were utilized. Precoated Matrigel and cells were added to the upper chambers. Serum‐free DMEM medium was added in 8‐μm pore Transwell plates (Corning, MA, USA). Next, cells in the upper chamber were removed using a cotton swab. Cells in the lower chamber were fixed using methanol and stained with 1% crystal violet.

### 2.8 qRT‐PCR

TRIzol regent (Thermo Fisher Scientific) was utilized to extract total RNA, which was preserved at −80°C. RNA concentrations were assessed using a biological spectrometer. cDNA synthesis was carried out using the PrimerScript RT Master kit from Takara Biotechnology (Dalian, China). FOXD2‐AS1, miR‐206, and MAP3K1 levels were quantified using RT‐qPCR by employing SYBR Green PCR Master mix (Roche, Mannheim, Germany) and an Applied Biosystems 7900 Real‐Time PCR System. Changes in FOXD2‐AS1 levels in the serum were calculated using 2^−ΔCt^. Relative gene expression was calculated using 2^−ΔΔCt^. The related primer sequences were shown in Table 1.

**TABLE 1:** Primers for real‐time PCR

Genes	Forward (5′‐3′)	Reverse (5′‐3′)
GAPDH	AAGAAGGTGGTGAAGCAGGC	GTCAAAGGTGGAGGAGTGGG
FOXD2‐AS1	TGGACCTAGCTGCAGCTCCA	AGTTGAAGGTGCACACACTG
U6	CTCGCTTCGGCAGCACA	AACGCTTCACGAATTTGCGT
MiR‐206	CCACACACTTCCTTACATTCCA	GCGAGCACAGAATTAATACGAC
MAP3K1	TAGATCACATGCCCACCACAT	AGCCCTTAAGCCCTGGAAGTC

### 2.9 Western blot

Proteins were separated using a 10% SDS‐PAGE gel and then, the proteins were transferred to PVDF membranes (Bio‐Rad). The PVDF membranes were washed using TBST containing 5% nonfat milk for 1 hours and then with antibodies against MAP3K1 and GAPDH (1:1000, CST, MA, USA) overnight at 4°C. The next day, secondary antibodies (1:2000, CST, MA, USA) were incubated with the membranes for 2 hours. ECL solution (Thermo Fisher Scientific) was used to develop the protein bands.

### 2.10 Luciferase assay

WT and MUT MAP3K1 were subcloned into pMIR‐GLO™ luciferase vectors (Promega). WT and MUT MAP3K1 (pMIR‐MAP3K1‐WT and pMIR‐MAP3K1‐MUT) sequences of luciferase reporter constructs were obtained from GeneChem (Shanghai, China). Cells were cotransfected with pMIR‐MAP3K1‐WT or pMIR‐MAP3K1‐MUT and miR‐129 or NC. Lipofectamine 3000 reagent (Thermo Fisher Scientific, Waltham, MA, USA) was used in all transfections. Luciferase assays were conducted with Dual‐Luciferase Reporter Assay System (Promega, Madison, WI, USA).

### 2.11 RNA immunoprecipitation assay

RNA immunoprecipitation (RIP) was conducted using the Magna RIP™ RNA‐Binding Protein Immunoprecipitation Kit (Millipore). Cells were lysed in RIP lysis buffer. A quantity of 100 μL of cell extract was coimmunoprecipitated in RIP buffer with magnetic beads conjugated to an anti‐Ago2 antibody or normal mouse IgG.

### 2.12 RNA pull‐down experiment

The purified RNAs were biotin‐labeled using the Pierce RNA 3’End Desthiobiotinylation Kit (Thermo Fisher Scientific). Cell lysates were treated with miR‐206‐Bio, miR‐206‐Bio‐mut, or NC‐Bio, and magnetic beads.

### 2.13 Animal studies

Twelve nude mice (6‐8 weeks, male) were obtained from the Shanghai Experimental Animal Center of the Chinese Academy of Sciences and the mice were maintained at the Experimental Animal Centre in a pathogen‐free facility. HepG2 cells, in which FOXD2‐AS1 was stably silenced, were injected into the flank area subcutaneously. One month later, the mice were sacrificed by cervical dislocation. All experiments were approved and supervised by the Animal Welfare and Ethics Committee of Renmin Hospital of Wuhan University.

### 2.14 Statistical analysis

Statistical analysis was done using GraphPad Prism 6.0. Statistical tests consisted of Student's *t* tests and one‐way analysis of variance, where appropriate. Differences were considered statistically significant at *P* < .05.

## 3 RESULTS

### 3.1 FOXD2‐AS1 level is increased and miR‐206 expression is decreased in HCC

**FIGURE 1:**
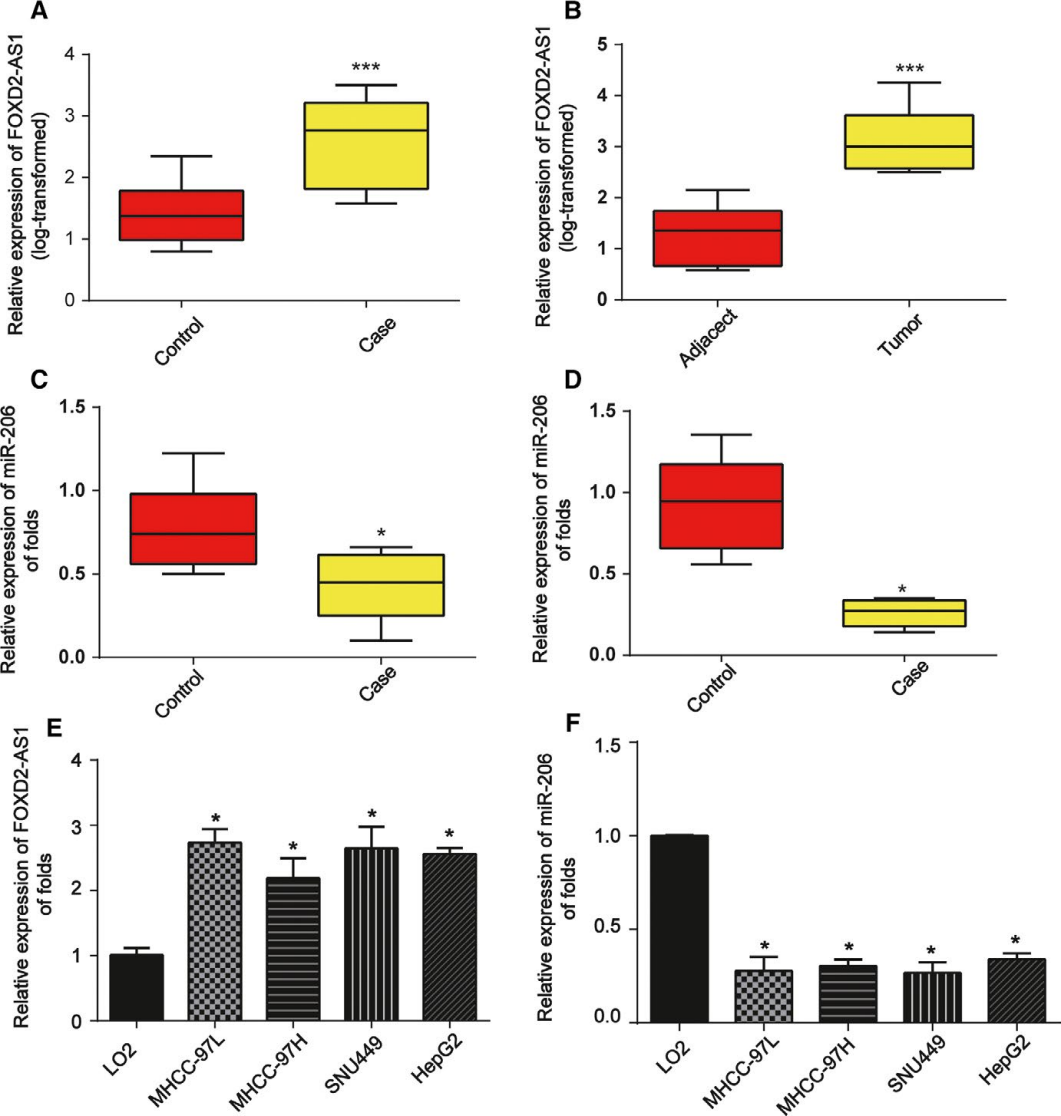
FOXD2‐AS1 is upregulated and miR‐206 is downregulated in HCC. A, Increased expression of FOXD2‐AS1 in HCC serum (n = 60) was detected in comparison to that from healthy controls (n = 60). B, Increased expression of FOXD2‐AS1 was detected in HCC tissues (n = 60) compared to the corresponding adjacent nontumor tissues (n = 60). C, Decreased expression of miR‐206 was detected in HCC serum (n = 60) compared to that from healthy controls (n = 60). D, Decreased expression of miR‐206 was detected in HCC tissues (n = 60) compared to the corresponding adjacent tissues (n = 60). E, FOXD2‐AS1 expression was assayed in HepG2, MHCC‐97L, MHCC‐97H, SNU449, and LO2 cells. GAPDH was used as a loading control. F, miR‐206 expression was assayed in HepG2, MHCC‐97L, MHCC‐97H, SNU449, and LO2 cells. U6 served as a loading control. Three independent experiments were performed. Error bars represented the mean ± SD of triplicate experiments (at least). *P < .05; ***P < .001

We first examined the level of FOXD2‐AS1 in the serum of HCC patients. FOXD2‐AS1 expression was markedly elevated in HCC patients compared to that in healthy controls (Figure 1A). Next, FOXD2‐AS1 expression was examined in 60 pairs of HCC tumors and adjacent nontumor tissues. Consistently, we observed that FOXD2‐AS1 was also significantly upregulated in tumor tissues (Figure 1B). In addition, miR‐206 expression was decreased in the serum of HCC patients and HCC tumor tissues compared to that of the controls (Figure 1C,D). We also examined the differences in FOXD2‐AS1 and miR‐206 expression in HCC cells compared to those in LO2 cells and observed that FOXD2‐AS1 expression was increased in HCC cells compared to that in LO2 cells (Figure 1E). In contrast, miR‐206 expression was downregulated in HCC cells (Figure 1F). We also analyzed the clinicopathological characteristics of the 60 HCC patients (Table 2). Analysis of the plasma samples of the patients indicated FOXD2‐AS1 and miR‐206 were negatively correlated with HCC carcinogenesis (Table 3).

**TABLE 2:** Clinicopathological relevance analysis of FOXD2‐AS1 in HCC patients

Feather	All patients	FOXD2‐AS1		*P* value
		Low expression (<median)	High expression (≥median)	
All cases	60	30	30	
Age, y				.665
<60	40	18	22	
≥60	20	9	11	
Gender				.539
Male	43	23	20	
Female	17	8	9	
Differentiation grade				.016
Well	28	19	9	
Moderate	11	5	6	
Poorly	21	5	16	
Tumor size (cm)				.000
≤5	32	20	12	
>5	28	9	19	
Tumor number				.185
Solitary	35	25	10	
Multiple	25	19	6	
Tumor capsular				.012
Incomplete	14	3	11	
Complete	46	28	18	
TNM stage (I:II:III)				.000
I	26	19	7	
II	12	7	5	
III	22	10	12	
Metastasis				.001
Yes	20	9	11	
No	40	22	18	

Note

Total data from 60 HCC patients were analyzed. For FOXD2‐AS1 expression, the median expression level was used as the cutoff. Data were analyzed by chi‐squared test. *P*‐value in bold indicated statistically significant.

**TABLE 3:** Clinicopathological information from HCC serum samples

Feather	All patients	FOXD2‐AS1		*P* value
		Low expression (<median)	High expression (≥median)	
All cases	60	30	30	
Age, y				.365
<60	32	12	20	
≥60	28	15	13	
Gender				.521
Male	45	20	25	
Female	15	8	7	
Differentiation grade				.015
Well	8	6	2	
Moderate	24	11	13	
Poorly	28	10	18	
Tumor size (cm)				.000
≤5	34	16	18	
>5	26	10	16	
Tumor number				.25
Solitary	42	21	21	
Multiple	18	6	12	
Tumor capsular				.02
Incomplete	20	14	6	
Complete	40	29	11	
TNM stage (I: II: III)				.000
I	32	20	12	
II	14	8	6	
III	12	2	10	
Metastasis				.001
Yes	17	4	13	
No	43	32	11	

Note

Total data were from 60 serum samples of HCC patients. For the expression of FOXD2‐AS1, median expression level was used as the cutoff. Data were analyzed using chi‐squared test. *P*‐value in bold was considered statistically significant.

### 3.2 Silencing FOXD2‐AS1 suppresses HCC growth, migration, and invasion

**FIGURE 2:**
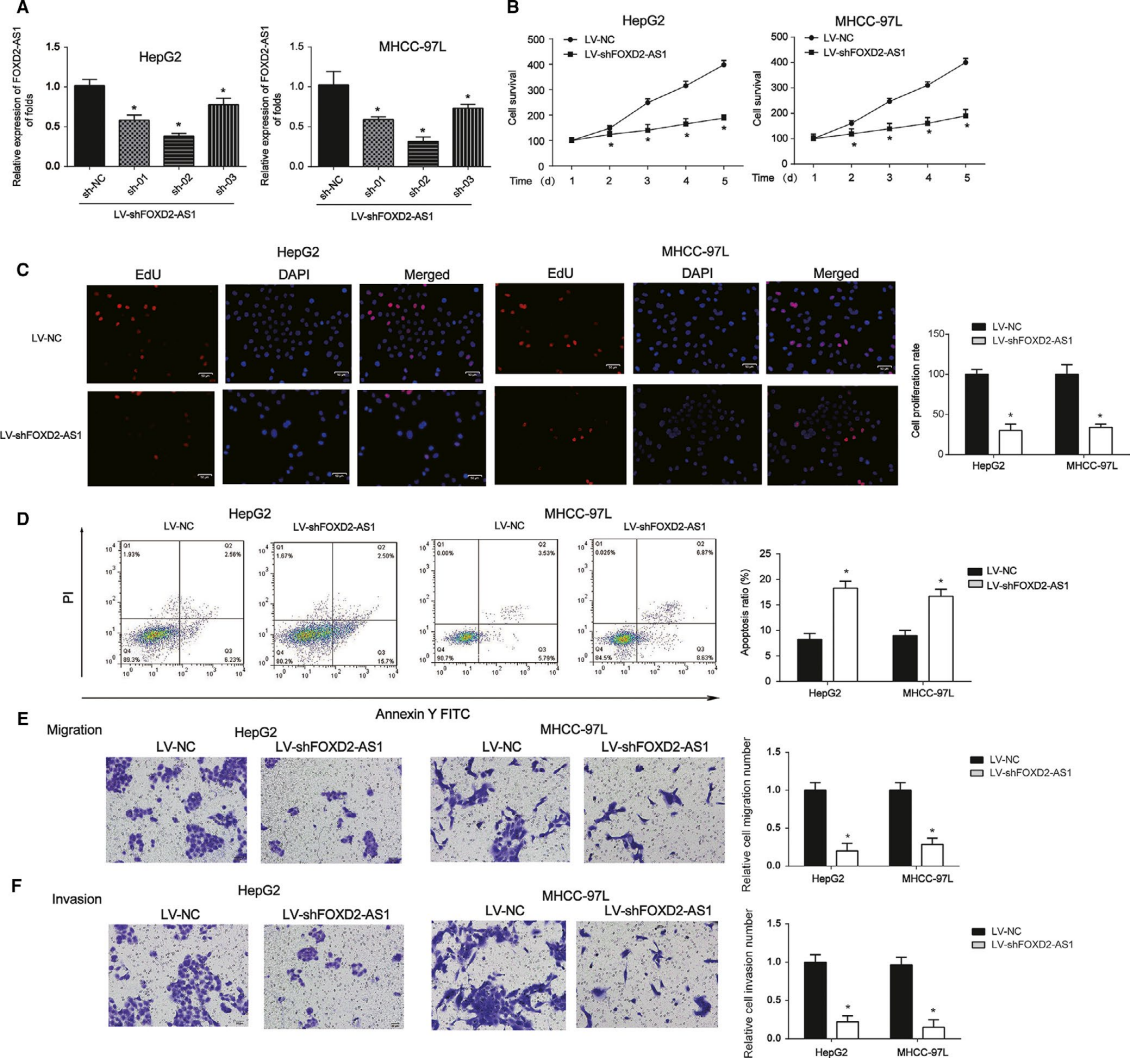
Effects of FOXD2‐AS1 downregulation on HCC cell growth, migration, and invasion. A, FOXD2‐AS1 expression was detected in HepG2 and MHCC‐97L cells. Cells were infected with control shRNA, sh01, sh02, or sh03‐shFOXD2‐AS1 for 48 h. B, CCK8 assay was performed to evaluate the effects of LV‐shFOXD2‐AS1 on cell survival in HepG2 and MHCC‐97L cells. C, EdU assay was used to assess the effects of LV‐shFOXD2‐AS1 on the proliferation of HepG2 and MHCC‐97L cells. D, Flow cytometry assay was used to analyze the effects of LV‐shFOXD2‐AS1 on the apoptosis of HepG2 and MHCC‐97L cells. E, Transwell migration assay was performed to test the effects of LV‐shFOXD2‐AS1 on the migration of HepG2 and MHCC‐97L cells. Cells were infected with LV‐shFOXD2‐AS1 for 48 h. F, Transwell migration assay was performed to test the effects of LV‐shFOXD2‐AS1 on the invasion of HepG2 and MHCC‐97L cells. Three independent experiments were performed. Error bars represented the mean ± SD of at least three independent experiments. *P < .05

Next, to determine whether FOXD2‐AS1 affected HCC cell proliferation, CCK8 assay was performed. Hepatocellular carcinoma cells were infected with sh‐NC, sh‐FOXD2‐AS1, sh01, sh02, or sh03 for 48 hours. qRT‐PCR analysis indicated that sh02 exhibited the strongest effect on FOXD2‐AS1 levels (Figure 2A). Hepatocellular carcinoma cell survival was repressed in response to decreased FOXD2‐AS1 expression in HCC cells (Figure 2B). EdU assays proved that cell proliferation was inhibited by LV‐shFOXD2‐AS1 (Figure 2C). In addition, the knockdown of FOXD2‐AS1 strongly triggered apoptosis of HepG2 and MHCC‐97L cells (Figure 2D). The migration and invasion capacity of HCC cells was restrained by LV‐shFOXD2‐AS1 (Figure 2E,F). These findings indicate that loss of FOXD2‐AS1 inhibits HCC cell growth, migration, and invasion.

### 3.3 MiR‐206 is a target of FOXD2‐AS1

**FIGURE 3:**
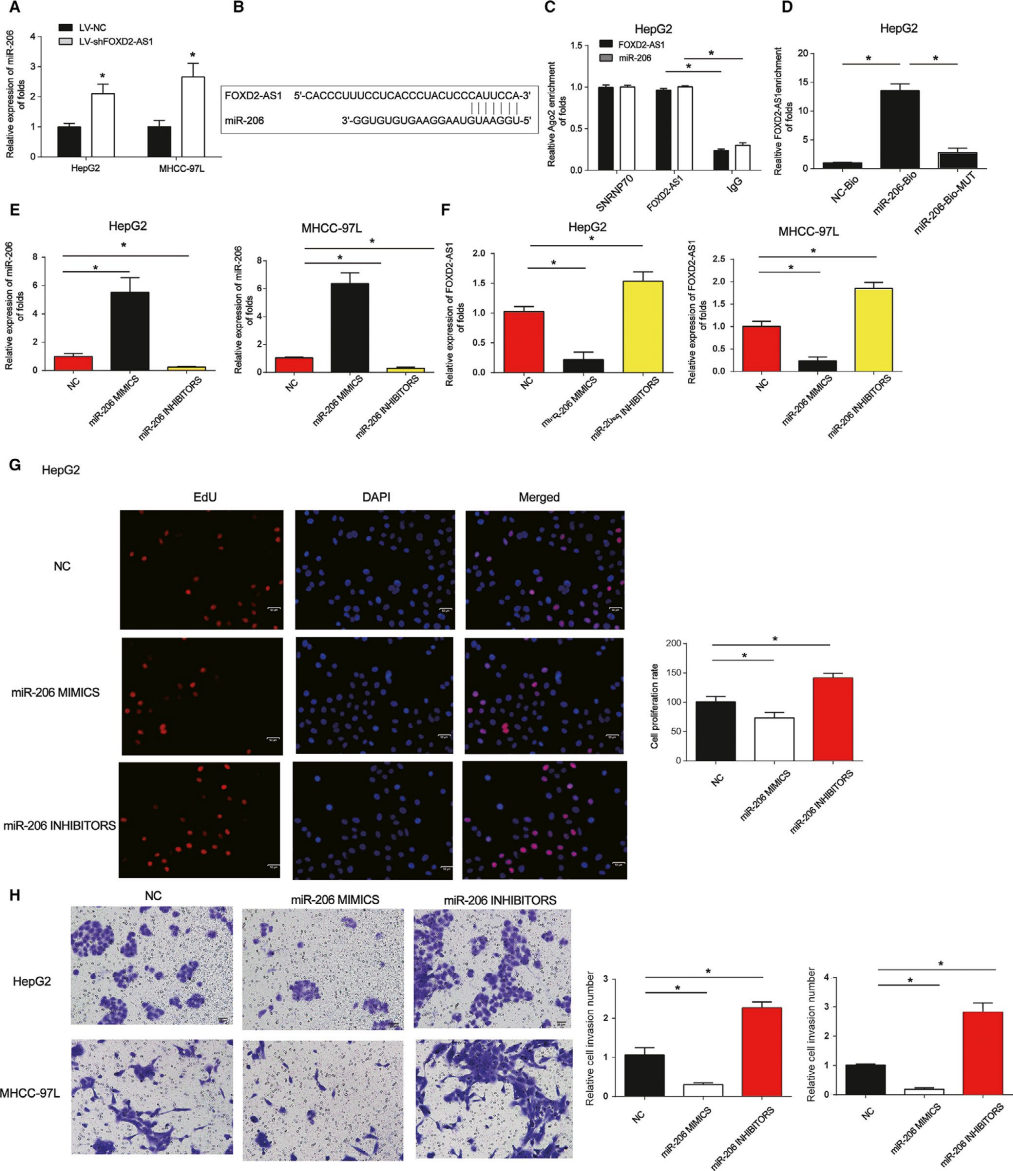
miR‐206 is a direct target of FOXD2‐AS1. A, miR‐206 expression was assayed in HepG2 and MHCC‐97L cells. Cells were infected with LV‐shFOXD2‐AS1 for 48 h. B, A schematic showed the interaction between miR‐206 and FOXD2‐AS1. C, The interaction between FOXD2‐AS1 and Ago2 was detected by RIP assays. Cellular lysates were immunoprecipitated using an Ago2 or IgG antibody. FOXD2‐AS1 expression was detected using qRT‐PCR. SNRNP70 acted as a positive control. D, RNA pull‐down assays implied a direct interaction between miR‐206 and FOXD2‐AS1. RNA from cellular lysates was precipitated using a biotinylated control (NC‐Bio), miR‐206 (miR‐206‐Bio), or a miR‐206 probe containing mutations in the FOXD2‐AS1‐binding site (miR‐206‐Bio‐mut). E, miR‐206 expression was detected in HepG2 and MHCC‐97L cells. Cells were transfected with miR‐206 mimics, inhibitors, or their NCs for 48 h. F, FOXD2‐AS1 expression was detected in HepG2 and MHCC‐97L cells. G, Effects of miR‐206 was evaluated on the proliferation of HepG2 cells. H, Effects of miR‐206 was assayed on the invasion of HepG2 and MHCC‐97L cells. Three independent experiments were performed. Error bars represented the mean ± SD of at least three independent experiments. *P < .05

To examine the interaction between FOXD2‐AS1 and miR‐206, HCC cells were infected with LV‐shFOXD2‐AS1. miR‐206 expression was elevated in response to LV‐shFOXD2‐AS1 (Figure 3A). A schematic of the interaction between miR‐206 and FOXD2‐AS1 is indicated in Figure 3B. In addition, RIP assays evidenced that FOXD2‐AS1 and miR‐206 were abundant in Ago2 pellets (Figure 3C). Moreover, RNA pull‐down assays showed an interaction between FOXD2‐AS1 and miR‐206 (Figure 3D). These findings imply that miR‐206 is a target of FOXD2‐AS1. We then tested whether miR‐206 overexpression could regulate HCC development. MHCC‐97L and HepG2 cells were transfected with miR‐206 mimics or inhibitors. miR‐206 level was increased by its mimics and inhibited by the inhibitors (Figure 3E). Interestingly, miR‐206 mimics significantly suppressed FOXD2‐AS1 expression, whereas miR‐206 inhibitors induced its expression (Figure 3F). Furthermore, miR‐206 mimics repressed HCC cell proliferation, while miR‐206 inhibitors promoted proliferation (Figure 3G). miR‐206 mimics repressed the invasion of MHCC‐97L and Hep3B cells, while miR‐206 promoted cell invasion (Figure 3H). These data suggest that miR‐206 modulates HCC development.

### 3.4 MAP3K1 is a target of miR‐206

**FIGURE 4:**
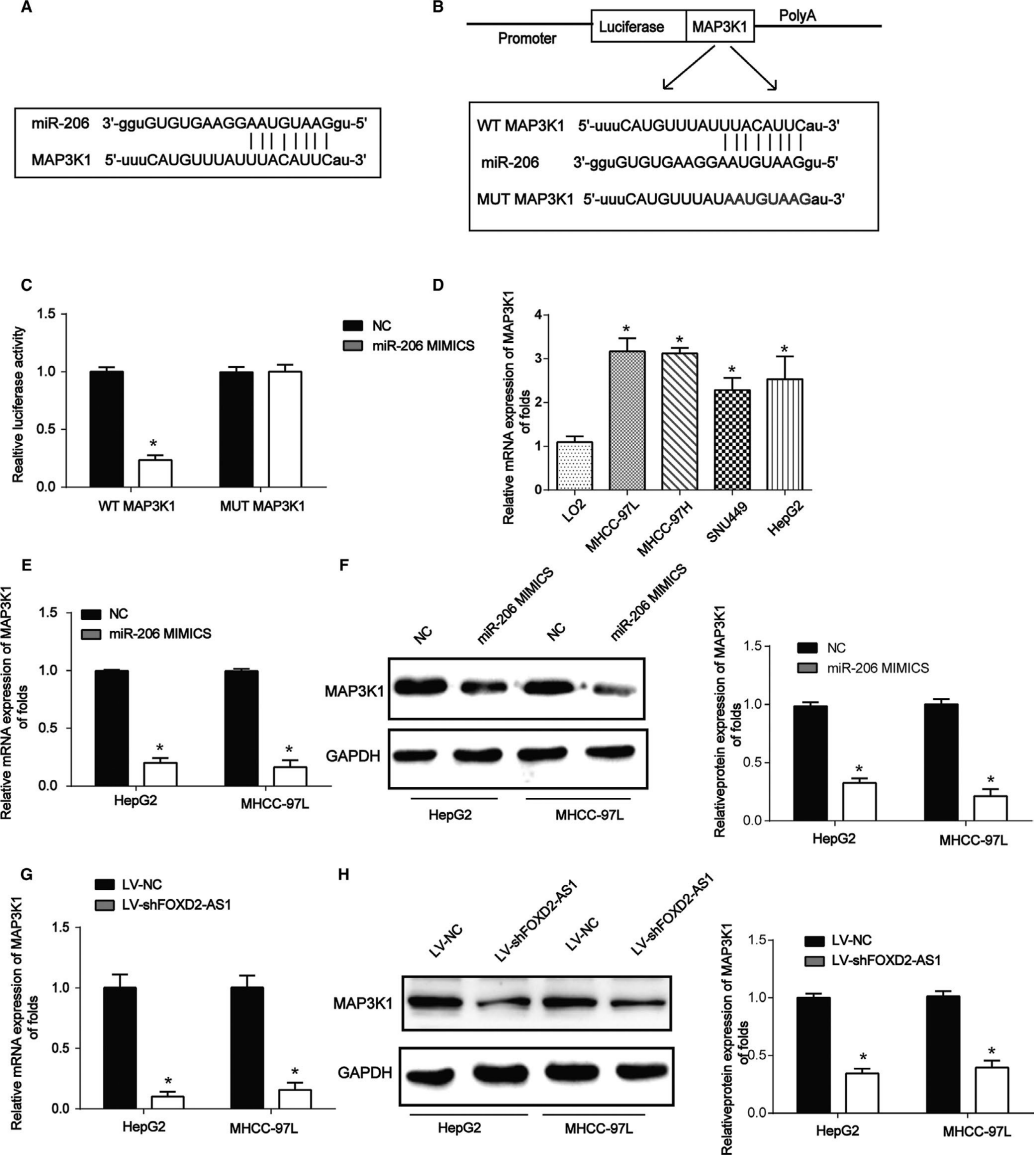
MAP3K1 is a direct target of miR‐206. A, A schematic showed the interaction between MAP3K1 and miR‐206. B, Luciferase reporter constructs containing the wild‐type (WT‐MAP3K1) or mutant MAP3K1 (MUT‐MAP3K1) sequence. C, WT‐MAP3K1 or MUT‐MAP3K1 sequence was co‐transfected with miR‐206 mimics or NCs into HEK‐293T cells. D, Expression of MAP3K1 mRNA was detected in HCC and LO2 cells. E, Expression of MAP3K1 mRNA was assayed in HCC cells. Cells were transfected with miR‐206 mimics or their NCs for 48 h. F, Expression of MAP3K1 protein was detected in HCC cells. G, Expression of MAP3K1 mRNA was detected in HCC cells. Cells were infected with LV‐shFOXD2‐AS1 for 48 h. H, Expression of MAP3K1 protein was assayed in HCC cells. Three independent experiments were performed. Error bars represented the mean ± SD of at least three independent experiments. *P < .05

MAP3K1 was also predicted as a target of miR‐206, and a schematic of their interaction was described in Figure 4A. WT‐MAP3K1 and MUT‐MAP3K1 were shown in Figure 4B. Cotransfection of WT‐MAP3K1 with miR‐206 markedly suppressed reporter activity (Figure 4C). MAP3K1 mRNA expression was strongly induced in HCC cells compared to that in LO2 cells (Figure 4D). We then showed that MAP3K1 was inhibited by miR‐206 mimics (Figure 4E,F). Next, as shown in Figure 4G,H MAP3K1 expression was inhibited by LV‐shFOXD2‐AS1 infection in HCC cells. These data suggest that FOXD2‐AS1 can modulate HCC progression by targeting miR‐206/MAP3K1.

### 3.5 Silencing of FOXD2‐AS1 inhibits HCC progression in vivo

**FIGURE 5:**
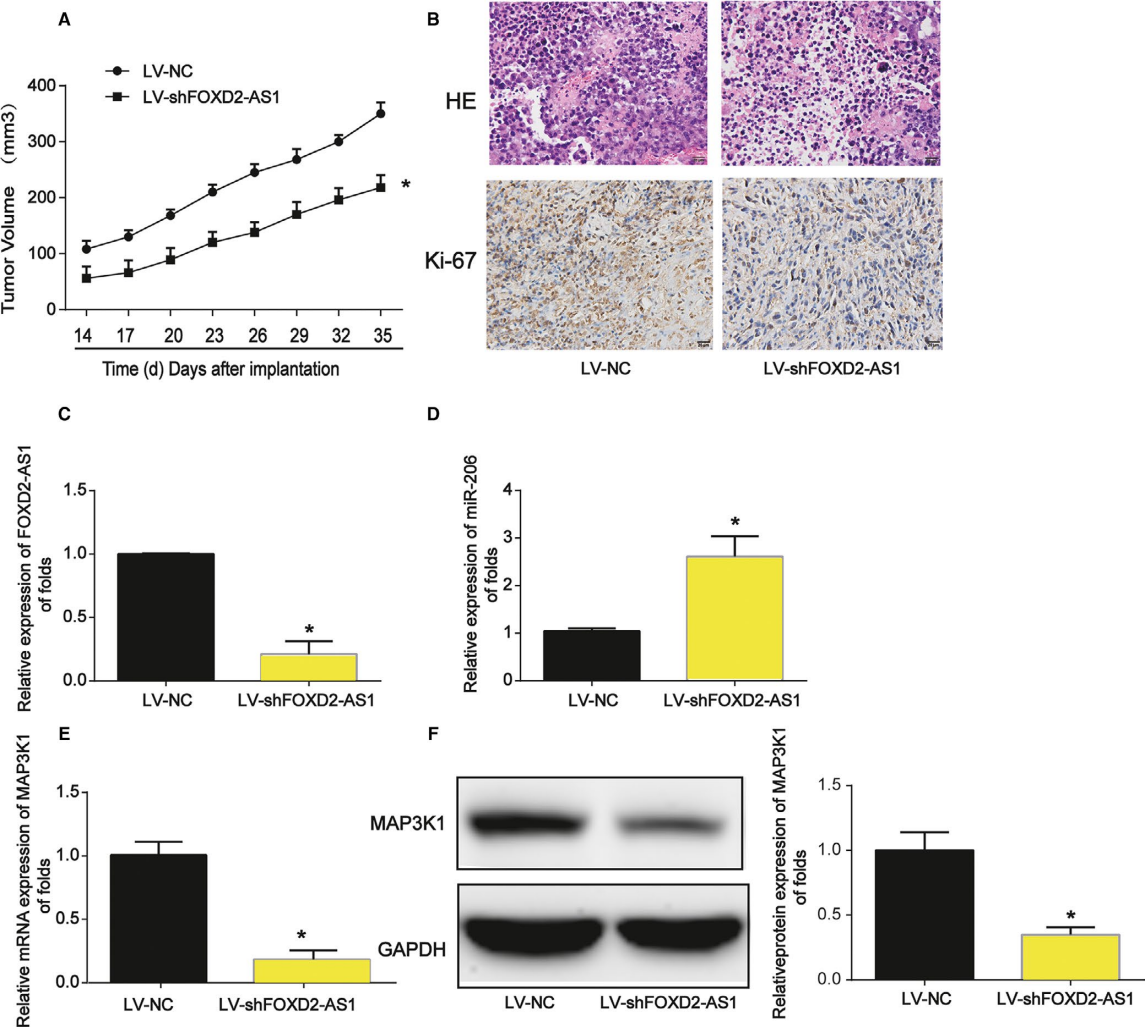
Downregulation of FOXD2‐AS1 inhibits HCC progression in vivo. Twelve 6‐ to 8‐week‐old male BALB/c nude mice were injected with HepG2 cells (n = 6) or LV‐shFOXD2‐AS1–infected HepG2 cells (n = 6). A, Tumor size changed in a time‐dependent manner. B, H&E staining and immunohistochemistry staining of Ki‐67 in tumor tissues. C, qRT‐PCR quantification of FOXD2‐AS1 expression was performed in mouse tumor tissues. D, Expression of miR‐206 was detected in mouse tumor tissues. U6 served as a loading control. E, Expression of MAP3K1 mRNA was detected in mouse tumor tissues. F, Expression of MAP3K1 protein was detected in mouse tumor tissues. Three independent experiments were carried out. Error bars represented the mean ± SD of at least three independent experiments. *P < .05

Subsequently, an in vivo assay was employed to confirm the effect of FOXD2‐AS1 on HCC progression. Six mice were injected in the front dorsum with parental HepG2 cells and the other six were injected with HepG2 cells infected with LV‐shFOXD2‐AS1. Inhibition of FOXD2‐AS1 profoundly inhibited tumor growth (Figure 5A). HE staining and IHC showed Ki‐67 was depressed by LV‐shFOXD2‐AS1 (Figure 5B). Loss of FOXD2‐AS1 repressed MAP3K1 through sponging miR‐206 (Figure 5C‐F). Thus, the downregulation of FOXD2‐AS1 inhibits HCC development in vivo.

### 3.6 FOXD2‐AS1 is a biomarker for HCC diagnostic prediction

**FIGURE 6:**
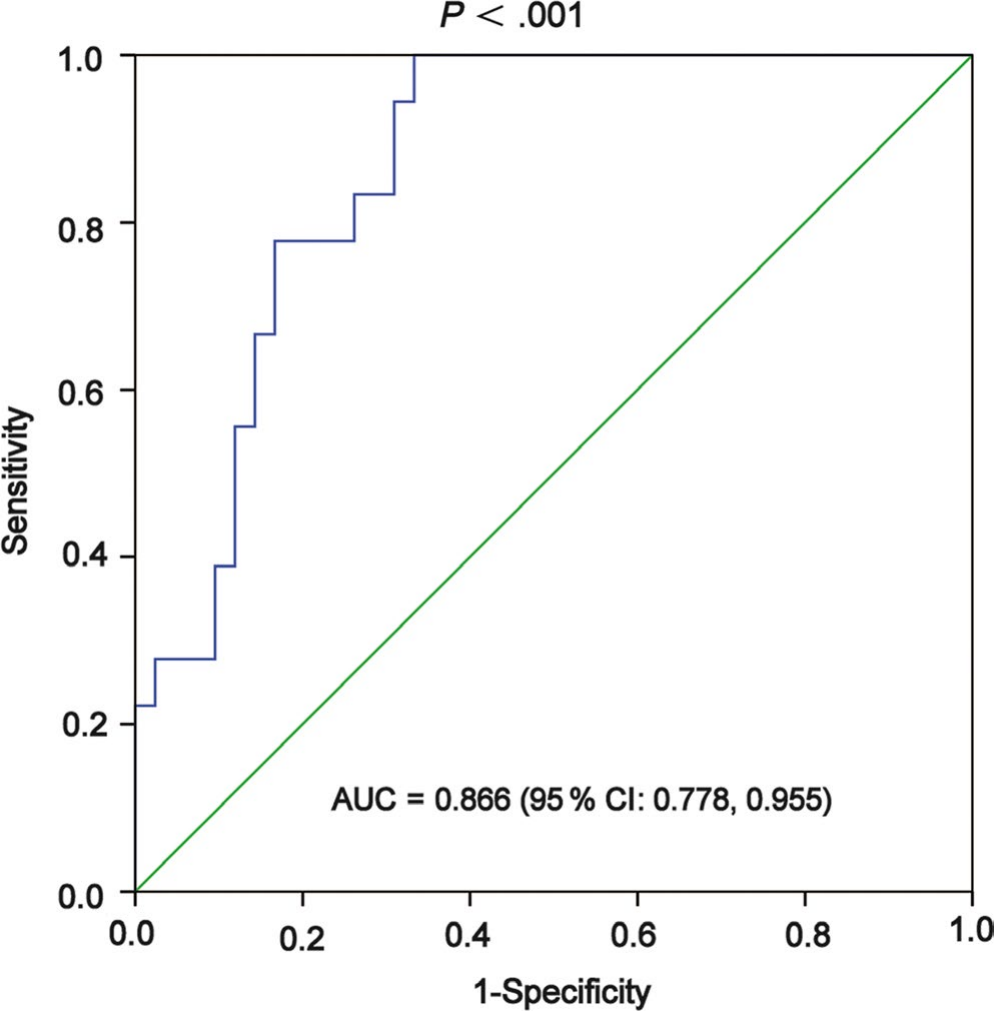
FOXD2‐AS1 is a biomarker for HCC diagnostic prediction. ROC curve analysis was performed to evaluate the diagnostic value of FOXD2‐AS1 in HCC

Kaplan‐Meier analysis was conducted to identify the correlation between FOXD2‐AS1 and HCC patient survival. We detected the expression level of FOXD2‐AS1 in HCC serum in all patients. FOXD2‐AS1 were high expressed in HCC with an AUC of 0.866 (Figure 6). These results suggest that FOXD2‐AS1 is an appropriate biomarker for HCC diagnostic prediction.

## 4 DISCUSSION

Many lncRNAs have been reported as effective cancer biomarkers. In this study, we found that in HCC cells, lncRNA FOXD2‐AS1 expression was significantly elevated, whereas miR‐206 expression was downregulated. Additionally, silencing FOXD2‐AS1 suppressed HCC progression, and miR‐206 mimics repressed HCC progression in vitro. FOXD2‐AS1 negatively regulated miR‐206 expression, and MAP3K1 was predicted a target of miR‐206.

FOXD2‐AS1 acts as an oncogene in various cancers. FOXD2‐AS1 promoted nasopharyngeal cancer by regulating miR‐363‐5p^25^ and acted as an oncogene in colorectal cancer by modulating Notch.^26^ Roles for FOXD2‐AS1 in HCC progression have recently been reported. For instance, FOXD2‐AS1 acted as a ceRNA against miR‐150‐5p and reversed sorafenib resistance in HCC.^27^ FOXD2‐AS1 exerted an oncogenic role in HCC by silencing CDKN1B and EZH2.^28^ In addition, FOXD2‐AS1 can also contribute to HCC development by targeting miR‐206.^29^ Here, we validated the FOXD2‐AS1 level was increased in HCC cells and found that the knockdown of FOXD2‐AS1 markedly restrained HCC cell growth, migration, and invasion. FOXD2‐AS1 expression can be a novel biomarker to predict HCC diagnosis.

LncRNAs can act as microRNA sponges in several cancers. MALAT1 triggered the progression of HCC by sponging miR‐146b‐5p.^30^ XIST was able to modulate PTEN through sponging miR‐181a, which promoted HCC progression.^31^ ****Moreover, TUSC7 sponged miR‐10a in HCC.^32^ miR‐206 was predicted to be a FOXD2‐AS1 target in our study. In previous studies, miR‐206 has been reported to inhibit HCC by regulating CDK9.^33^ And miR‐206 overexpression can repress HCC development.^34^ Furthermore, we observed that FOXD2‐AS1 knockdown increased miR‐206 levels. A direct interaction between FOXD2‐AS1 and miR‐206 was established using RIP and RNA pull‐down assays.

MAPKs are significant mediators of cell physiology.^35^, ^36^ MAP3K1 belongs to the MAP3K family,^37^ and studies have shown that MAP3K1 impacted several cellular processes in tumors.^38^, ^39^ miR‐451 can inhibit esophageal carcinoma cell proliferation by suppressing MAP3K1.^40^ miR‐302 increased the sensitivity of breast cancer cells to adriamycin through targeting MAP3K1.^41^ MAP3K1 was a target of miR‐206, and interestingly, miR‐206 inhibited HCC development by targeting MAP3K1. The interaction between miR‐206 and MAP3K1 was validated using a dual luciferase reporter assay. We also found that FOXD2‐AS1 positively regulated MAP3K1 by sponging miR‐206. In the future, we will investigate the function of MAP3K1 in HCC in details.

Overall, our findings indicate that FOXD2‐AS1 plays an oncogenic role in HCC. The oncogenic function of FOXD2‐AS1 is mediated by its interaction with miR‐206 and MAP3K1. In addition, FOXD2‐AS1 can be used as a biomarker for HCC diagnosis.

## 5 CONFLICT OF INTEREST STATEMENT

The authors declare no conflict of interest.

## AUTHORSHIP STATEMENT

**Wenfang Xia** and **Wei Hu** designed the research; **Wei Hu**, **Hui Feng,** and **Xiaoyu Xu** performed the experiments; **Xin Huang** and **Xingyue Huang** collected the data; **Wenwei Chen** and **Lidan Hao** did the analysis; **Wei Hu** drafted the manuscript; **Wenfang Xia** revised the manuscript. All authors approved the final manuscript.

## ETHICAL APPROVAL STATEMENT

This study was approved by the Medical Ethics Committee of Renmin Hospital of Wuhan University, and all experiments were approved and supervised by the Animal Welfare and Ethics Committee of Renmin Hospital of Wuhan University.

## Data Availability

All data are available upon request.
